# Supplementary figures and images for: Vaginal microbiome of women with adenomyosis: A case-control study
Source: PLoS One. 2022 Feb 16;17(2):e0263283. doi: 10.1371/journal.pone.0263283 (PMC8849446; doi:10.1371/journal.pone.0263283)

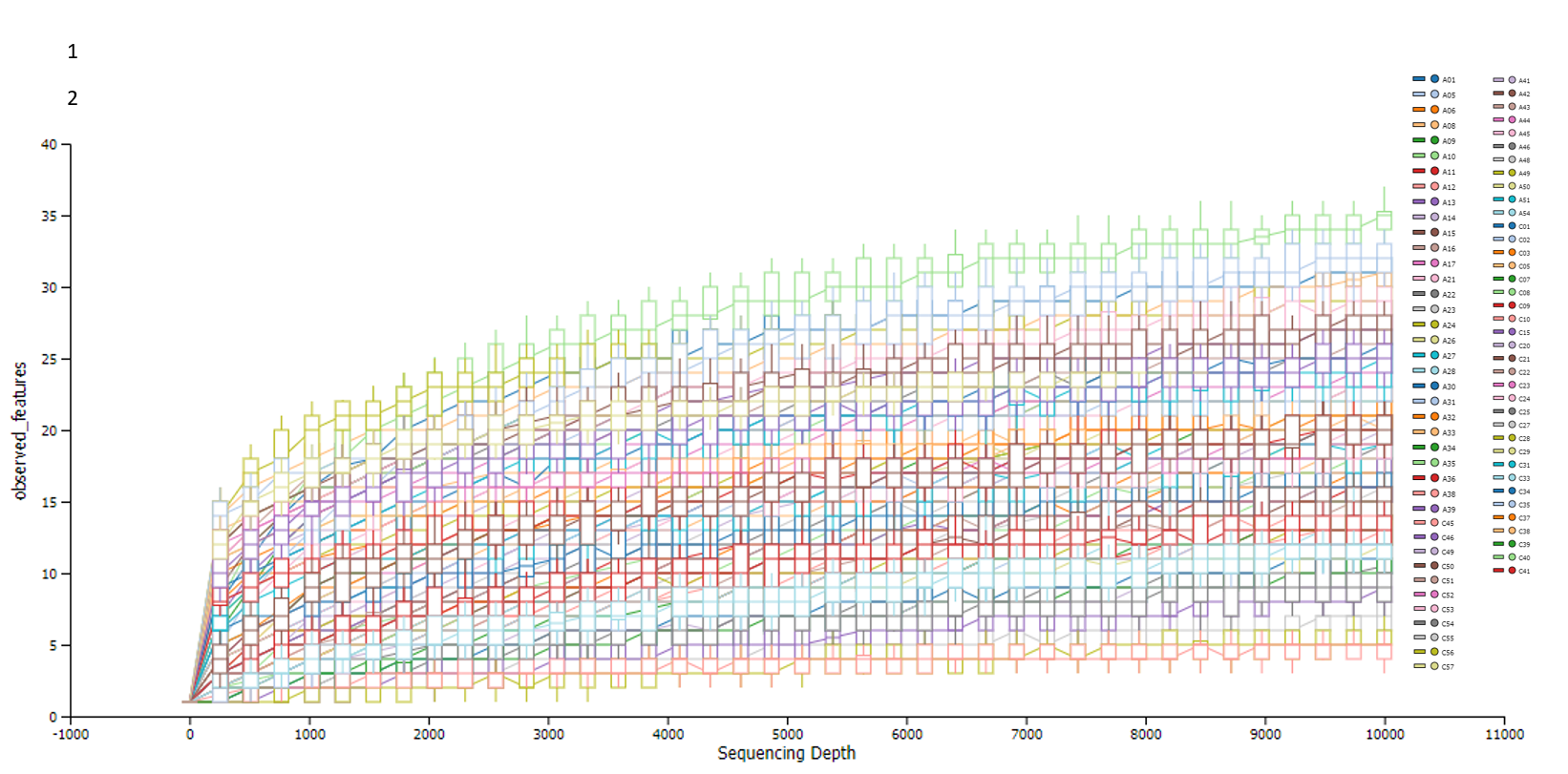

Supplement: S1 Fig — (TIF) [file pone.0263283.s001.tif]

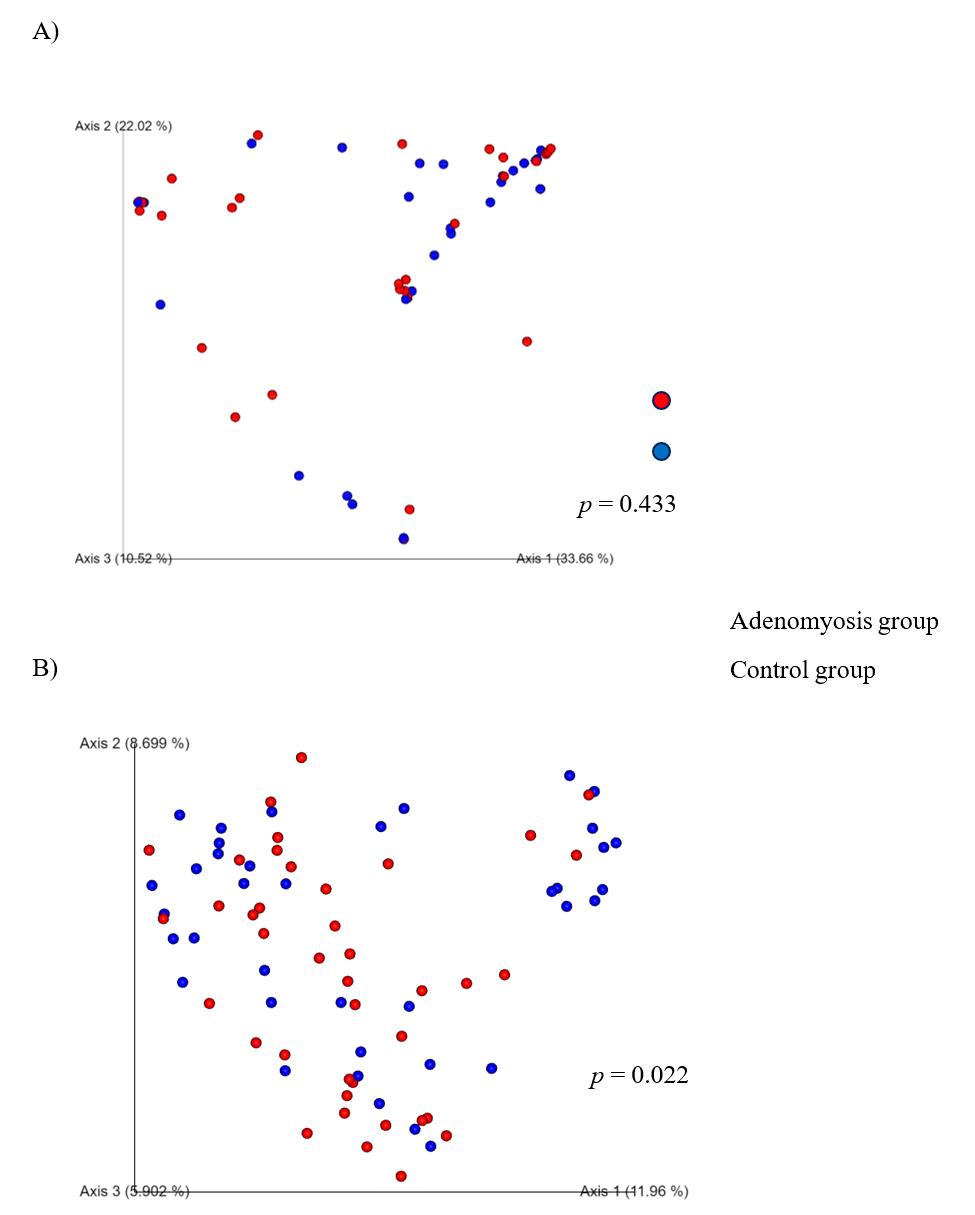

Supplement: S2 Fig — Beta diversity analysis of vaginal microbial in women with and without adenomyosis showed no significant difference by A) Bray-Curtis dissimilarities (abundant weighted distance) and B) Jaccard index (the presence or absence of operational taxonomic units [OTUs]). (TIF) [file pone.0263283.s002.tif]
