# Supplementary material for: Vaginal microbiome of women with adenomyosis: A case-control study
Source: PLoS One. 2022 Feb 16;17(2):e0263283. doi: 10.1371/journal.pone.0263283 (PMC8849446; doi:10.1371/journal.pone.0263283)
Supplement: S1 Table — (DOCX) [file pone.0263283.s003.docx]

**
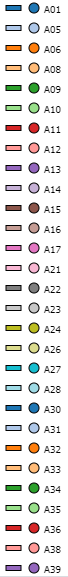

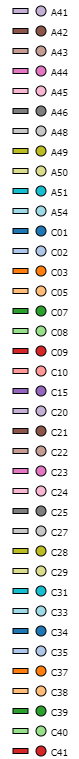

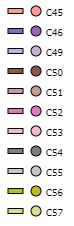
Supporting information**

|  | N (%) |
| --- | --- |
| **Symptoms**  **-** Pelvic pain  **-** Abnormal uterine bleeding  **-** Pressure symptom  **-** Infertility  **-** Asymptomatic | 25 (62.5)  14 (35.0)  3 (7.5)  4 (10.0)  2 (5.0) |
| **Onset of symptoms**  **-** ≤ 2 years  **-** > 2 years | 23 (57.5)  17 (42.5) |
| **Type of adenomyosis**  **-** Diffuse  **-** Focal | 32 (80.0)  9 (22.5) |
| **Uterine size**  **-** ≤ 8 weeks size  **-** > 8 weeks size | 25 (62.5)  15 (37.5) |
| **Other gynecologic diseases**  **-** Uterine fibroid  **-** Ovarian endometriosis  **-** Pelvic endometriosis  **-** Endometrial polyps  **-** None | 12 (30.0)  20 (50.0)  4 (10.0)  5 (12.5)  2 (5.0) |

**Table S1:** Symptoms, characteristics of adenomyosis and other types of gynecological diseases in the adenomyosis group.
