# Supplementary material for: Vaginal microbiome of women with adenomyosis: A case-control study
Source: PLoS One. 2022 Feb 16;17(2):e0263283. doi: 10.1371/journal.pone.0263283 (PMC8849446; doi:10.1371/journal.pone.0263283)
Supplement: Attachment — Submitted filename: Responses to reviewers.docx [file pone.0263283.s010.docx]

September 9^th^ , 2021

Dear Professors,

Editors-in-Chief,

Thank you very much. We deeply appreciate for considering our paper entitled “Vaginal Microbiome of Women with Adenomyosis: A Case-Control Study.” We have made all corrections as suggested by the reviewer and editor. The corrections are as follow in the manuscripts according to our responses to the editor’s comments as listed below.

Review Comments to the Author

Please use the space provided to explain your answers to the questions above. You may also include additional comments for the author, including concerns about dual publication, research ethics, or publication ethics. (Please upload your review as an attachment if it exceeds 20,000 characters)

Reviewer #1: The study is original and the experiments, statistics, and other analyses are performed to a high technical standard and are described in sufficient detail. I think the discussion and conclusion can be improved. And the paragraph (lines 296-303) needs to done again, read Chen's study again.

Our response:

Thank you so much for looking over our manuscript and gave the informative suggestions.

We are sorry for the mistake.

We went through the Chen C and Chen S studies. We re-wrote the sentences as shown in page 15 line 295-302. (yellow highlight)

Reviewer #2: The authors conducted an interesting study to evaluate differences in the vaginal microbiome of women with and without adenomyosis. The authors had well phenotyped cases, although the analysis of the vaginal microbiome was superficial relative to other vaginal microbiome studies. The authors only presented aggregate results and did not include any species level differences. This is not worthy given that some Lactobacillus species are thought to be more protective of reproductive health than others, notably L. crispatus. I think the study would be more comparable to other studies evaluating the vaginal microbiome with more complete bioinformatic processing of the sequencing data.

Our response:

Thank you so much for reviewing our manuscript and suggest us many good idea.

We agree with you. We re-analyzed the data which included using other database and also species analysis. We also compared the outcomes with other papers as suggestion.

There were also some concerns with some of the statements made throughout the manuscript. Some specific concerns are listed below.

This statement on page 6 line 92 needs to be re-worded or clarified:

"Lactobacillus that have been detected in the vagina, only one or two species are dominant in normal host vagina [13]." The sentence reads like only one or 2 species of Lactobacillus are present ever in the vagina instead of that it is common for women to have their vaginal microbiome composed of largely one lactobacillus species.

Our response:

We made a correction as suggested on page 6, line 93-96.

Line 96, change invader to pathogens.

Our response:

We made a correction as suggested on page 6, line 98.

Please clarify this statement pg 7 line 101, the meaning is unclear:

“The healthy vaginal flora combines numerous bacterial species in valid proportion with Lactobacilli”

Our response:

We made a correction as suggested on page 6 line 103. “The healthy vaginal flora combines numerous bacterial species with a certain large proportion of *Lactobacilli”*

Pg 7 line 103, what do you mean by dysbiosis? Non-lactobacillus predominance? Different from the women’s original baseline microbiome? Some women have diverse microbiota with no known pathology or symptoms.

Our response:

Thank you so much for this knowledge.

We made a correction on page 6 line 106. “Alteration of vaginal microbiota”

Please clarify which study you are referring to here pg 8 line 115-117, there is no citation & the Chen study the microbiomes were so different for these women, that women with the condition could be identified by the microbiome from any site along the reproductive tract. Is this the study (ref 22 in your list) to which you are referring? : “Although a previous study reported alterations of the bacterial microbiome in the reproductive tract of adenomyosis women, it focused directly on the correlation between vaginal microbiota composition and adenomyosis and was very limited.”

Our response:

We are sorry for the confusion. We rewrote and cited 3 papers, Chen C, Chen S and Chao on page

7 line 117-122.

Why did the authors use Greengenes to classify the sequences? That database is no longer maintained and hasn’t been updated since 2013. Assigning taxonomy with RDP, or another database that is still maintained would be more accurate.

Additionally, why were the analysis primarily completed at the phylum and genus level? Given that species level differences are important related to vaginal health outcomes, this should be done and has been consistently done in recent vaginal microbiome literature – particularly speciation of Lactobacillus. Some examples for reference:
Brooks JP, Buck GA, Chen G, et al. Changes in vaginal community state types reflect major shifts in the microbiome. Microb Ecol Health Dis. 2017;28(1):1303265. doi:10.1080/16512235.2017.1303265

Fettweis JM, Serrano MG, Brooks JLPL, et al. The vaginal microbiome and preterm birth. Nat Med. 2019;25(6):1012-1021. doi:10.1038/s41591-019-0450-

Elovitz MA, Gajer P, Riis V, et al. Cervicovaginal microbiota and local immune response modulate the risk of spontaneous preterm delivery. Nat Commun. 2019;10(1):1305. doi:10.1038/s41467-019-09285-9

Our response:

Thank you for your suggestions. We agreed with reviewer’s concern. Therefore, our analysis was improved by changing the database to SILVA 138.1. We decide to use SILVA database because it is larger than Greengenes and RDP. In addition, it was corrected for taxonomy and quality which because it might be more reliable than sequences from NCBI. The results showed the better resolution that most OTUs can be classified at genus level. In addition, it can improve the classification to species level for some OTUs.

Our analysis mainly focused on the only phylum and genus level because most of OTUs can be clearly classified to genus level due to the limitation of short-read 16S sequencing. [Jeong J, Yun K, Mun S, Chung WH, Choi SY, Nam YD, Lim MY, Hong CP, Park C, Ahn YJ, Han K. The effect of taxonomic classification by full-length 16S rRNA sequencing with a synthetic long-read technology. Sci Rep. 2021 Jan 18;11(1):1727. doi: 10.1038/s41598-020-80826-9.]

As reviewer suggestion, we adapted the bioinformatic methods to improve the classification of *Lactobacillus* species. Briefly, we extracted the reads of *Lactobacillus* and re-classified them by sklearn classifier against custom reference database of *Lactobacillus* species.

Megasphaera species are frequently present in women with bacterial vaginosis and diverse microbiota (versus lactobacillus dominant). It is interesting that this genus was more common in the control group and some discussion on this would be warranted/interesting, since the presence of these microbes has been associated with poor reproductive health outcomes.
Glascock AL, Jimenez NR, Boundy S, et al. Unique roles of vaginal Megasphaera phylotypes in reproductive health. bioRxiv. August 2020:2020.08.18.246819. doi:10.1101/2020.08.18.246819

Our response:

Thank you for your suggestions. We discussed about it as suggestion as shown in page 15, line 292-294, 306-308.

Why was Lactobacillus dominant classified in this way (pg 14 line 268)? This is not a standard way of classifying or evaluating the vaginal microbiome, which limits the ability to compare results to other studies.

Our response:

We agree with the reviewer. We correct the paragraph on page 16 line 325-328.

The paragraph beginning at line 304 on pg 16 seems out of place and grossly overstates the results. The authors results presented did not show women with adenomyosis have vaginal dysbiosis (lines 315-318), nor did they present any data indicating these women have inflammation.

Our response:

We agree with the reviewer. We misunderstood. Since we added discussion more for other issues and it might be redundant for having this paragraph, so we decide to delete the whole paragraph.

Are the data publicly available? The authors said the information is included with the supplementary information, but there is no data. The analysis can’t be reproduced or validated as submitted.

Our response:

We are so sorry. We uploaded it this time.

This citation is inaccurate, pg 14 line 264-265: “However, previous reports on the relationship between ethnicity and vaginal Lactobacillus abundance have been inconclusive [40-43].” The studies cited that there are differences in vaginal microbiome communities between ethnicities, although the reason for these differences are unknown. These differences are some of the most reproducible differences observed in the microbiome literature.

Our response:

We discussed more about the ethnicity and vaginal microbiome communities on page 16-17, line 325-353.

There is no figure 1?

Our response:

We are so sorry. It was a mistake while copying the figure.

.


Best regards,

Areepan Sophonsritsuk
