# Supplementary material for: Vaginal microbiome of women with adenomyosis: A case-control study
Source: PLoS One. 2022 Feb 16;17(2):e0263283. doi: 10.1371/journal.pone.0263283 (PMC8849446; doi:10.1371/journal.pone.0263283)
Supplement: Attachment — Submitted filename: Responses to reviewers.docx [file pone.0263283.s011.docx]

December 12^th^ , 2021

Dear Professors,

Editors-in-Chief,

Thank you very much. We deeply appreciate for considering our paper entitled “Vaginal Microbiome of Women with Adenomyosis: A Case-Control Study.” We have made all corrections as suggested by the reviewer and editor. The corrections are as follow in the manuscripts according to our responses to the editor’s comments as listed below.

Review Comments to the Author

Please use the space provided to explain your answers to the questions above. You may also include additional comments for the author, including concerns about dual publication, research ethics, or publication ethics. (Please upload your review as an attachment if it exceeds 20,000 characters)

Reviewer #1: I still think that the discussion can be improved, is missing a discussion of concepts between the disease and the results. The authors only can write: "is not known"; "is unknown as a cause or consequence of the disease"; "is unclear"; "we could not explain its existence". What is the relationship between immune dysregulation, adenomyosis and microbiota (results)? The authors can try to explain with concepts (literature) and the results.

Our response:

Thank you so much for looking over our manuscript. We appreciate for your suggestion.

We tried to explain the concepts between the disease and the results as shown in page 16 line 321 to page 17 line 334. (yellow highlight)

Reviewer #2:  I think the manuscript is much improved. I think one of the supporting data files has still not been uploaded (e.g., the information used for tables 1 and S1, nor CST assignments made for individuals). There are some places with some awkward/unusual phrasing, and I think a read through with an editor could improve these. Last request, please change the language throughout (e.g., abstract, intro, conclusion) that states a shift in microbiome is associated with adenomyosis. This was a cross-sectional study, without repeated measures and not change in microbiome over time was analyzed. The analysis conducted in this study does not support a shift, just that there may be different taxas present in people with vs without adenomyosis.

Our response:

Thank you so much for reviewing our manuscript again. We appreciate for your suggestion.

We uploaded the information used for tables 1 and S1 (S1 File. Data for Table 1 & S1.xlsx) , and CST assignments (S5 File. CST assignment).

We deleted some awkward paragraph/sentence.

We changed the language throughout (e.g., abstract, intro, conclusion) that states a shift in microbiome is associated with adenomyosis as suggestion on page 3, line 53-55, page 7, line 127-128, and page 20, line 399-400.

.


Best regards,

Areepan Sophonsritsuk
